# Acute Paraplegia Following Spinal Anesthesia in a Patient With Unrecognized Thoracic Ossification of the Posterior Longitudinal Ligament and Spinal Stenosis: A Case Report

**DOI:** 10.7759/cureus.68950

**Published:** 2024-09-08

**Authors:** Po-Wei Chen, Chih-Chang Chang, Tsung-Hsi Tu, Jau-Ching Wu, Wen-Cheng Huang

**Affiliations:** 1 Neurosurgery, Taipei Veterans General Hospital, Taipei, TWN; 2 Medicine, National Yang Ming Chiao Tung University, Taipei, TWN; 3 Biomedical Engineering, National Yang Ming Chiao Tung University, Taipei, TWN

**Keywords:** complication, ossification of posterior longitudinal ligament, spinal anesthesia, spinal coning, spinal stenosis

## Abstract

Spinal coning is a rare complication in spinal anesthesia that results in acute neurological deterioration. It occurs when a pre-existing spinal stenosis alters the CSF flow, creating a high-pressure area beneath the stenosis. The introduction of a needle into this relatively high-pressure area would cause a decrease in CSF pressure, exacerbating the spinal cord compression. This article reports a 50-year-old female who presented with urinary frequency for six months and was diagnosed with urethra spasm. Following spinal anesthesia, a botulinum toxin injection over the urethra was performed by a urologist. The patient did not recover from the anesthesia, which subsequently resulted in acute paraplegia status with bowel and bladder dysfunction. An MRI of her thoracic spine revealed ossification of the posterior longitudinal ligament with severe spinal stenosis. She received decompressive surgery and recovered well. Surgeons and anesthesiologists should be aware of patients who may have pre-existing spinal stenosis to avoid the use of spinal anesthesia and thus prevent spinal coning. Rapid neurological deterioration and severe disability warrant early aggressive surgical treatment for better recovery.

## Introduction

The herniation of neural elements between different anatomical spaces while performing lumbar puncture in response to increased intracranial pressure (IICP) is a well-defined concept. Collier proposed the term “pressure cone” to describe the phenomenon of cerebellar tonsillar herniation [1]. In patients with IICP, a significant pressure difference can be observed between the cranial and spinal compartments. Lowering the lumbar cerebrospinal fluid (CSF) pressure by a spinal CSF tap may aggravate the herniation through the foramen magnum. This “pressure cone” concept also applies to patients who have a spinal block caused by various pathologies [2]. A lumbar puncture can initiate neurological deterioration when CSF is withdrawn below the level of the spinal block. The compression resulting from a spinal block after CSF removal is referred to as “spinal coning.” Here, we present an unusual case with an undetected spinal block caused by ossification of the posterior longitudinal ligament (OPLL) who developed acute paraplegia after spinal anesthesia.

## Case presentation

**Figure 1:**
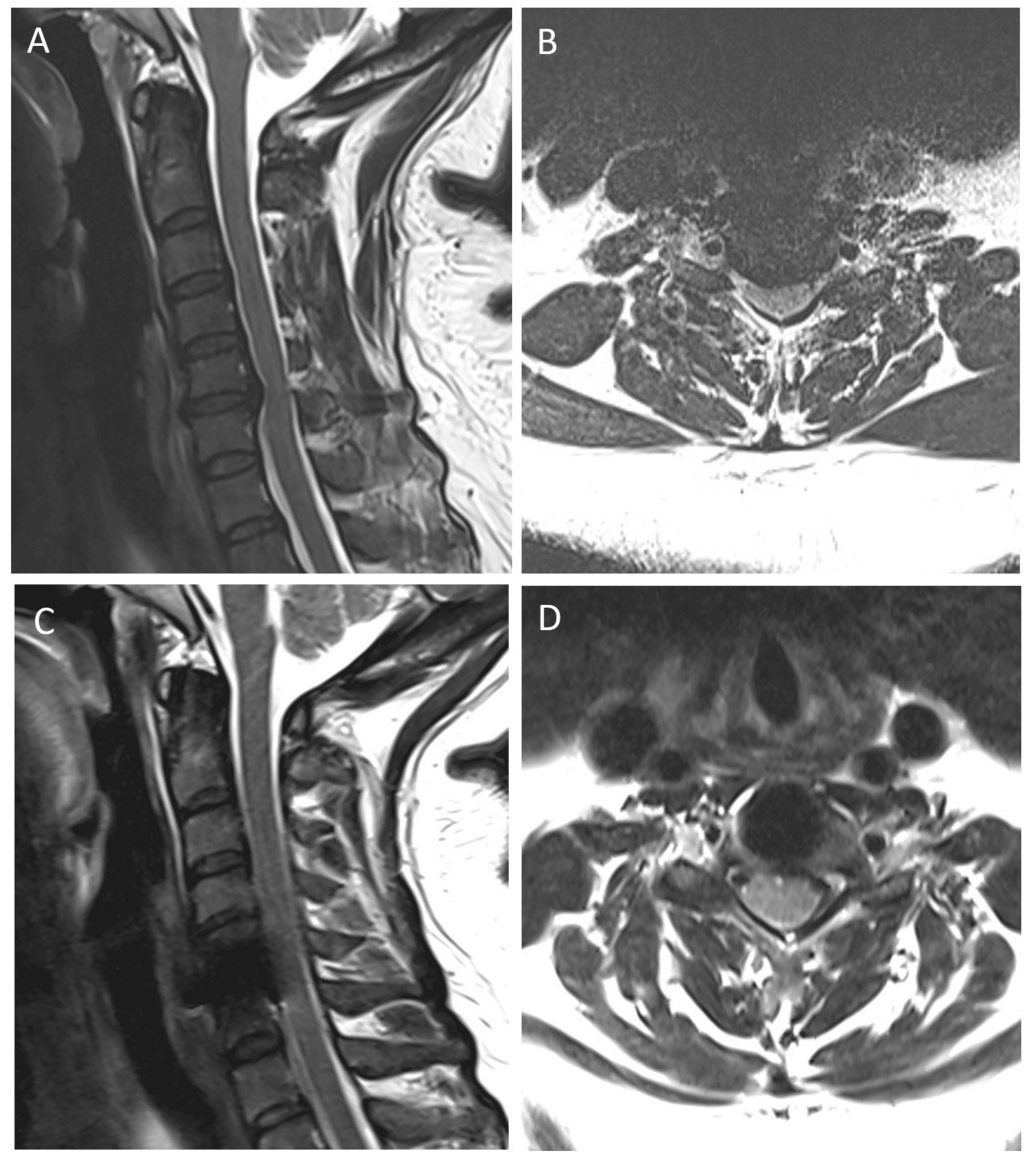
Pre- (A and B) and postoperative (C and D) T2-weighted MRI of the cervical spine Spinal cord compression with disc herniation over the C5–6 level was well decompressed. MRI: magnetic resonance imaging

The patient was a 50-year-old female with a history encompassing hypertension, type II diabetes mellitus, and a herniated vertebral disc at the C5-6 level, accompanied by the presence of mild segmental OPLL. The patient underwent C5-6 cervical disc arthroplasty two years before the current event (Figure 1).

Before this incident, the patient displayed ambulatory ability without experiencing any weakness or numbness in her four limbs. However, over six months, the patient experienced urinary frequency and was diagnosed with urethral spasm. Before the surgery, the patient showed no symptoms of myelopathy with full muscle power (Grade 5/5) over the bilateral limbs.

**Figure 2:**
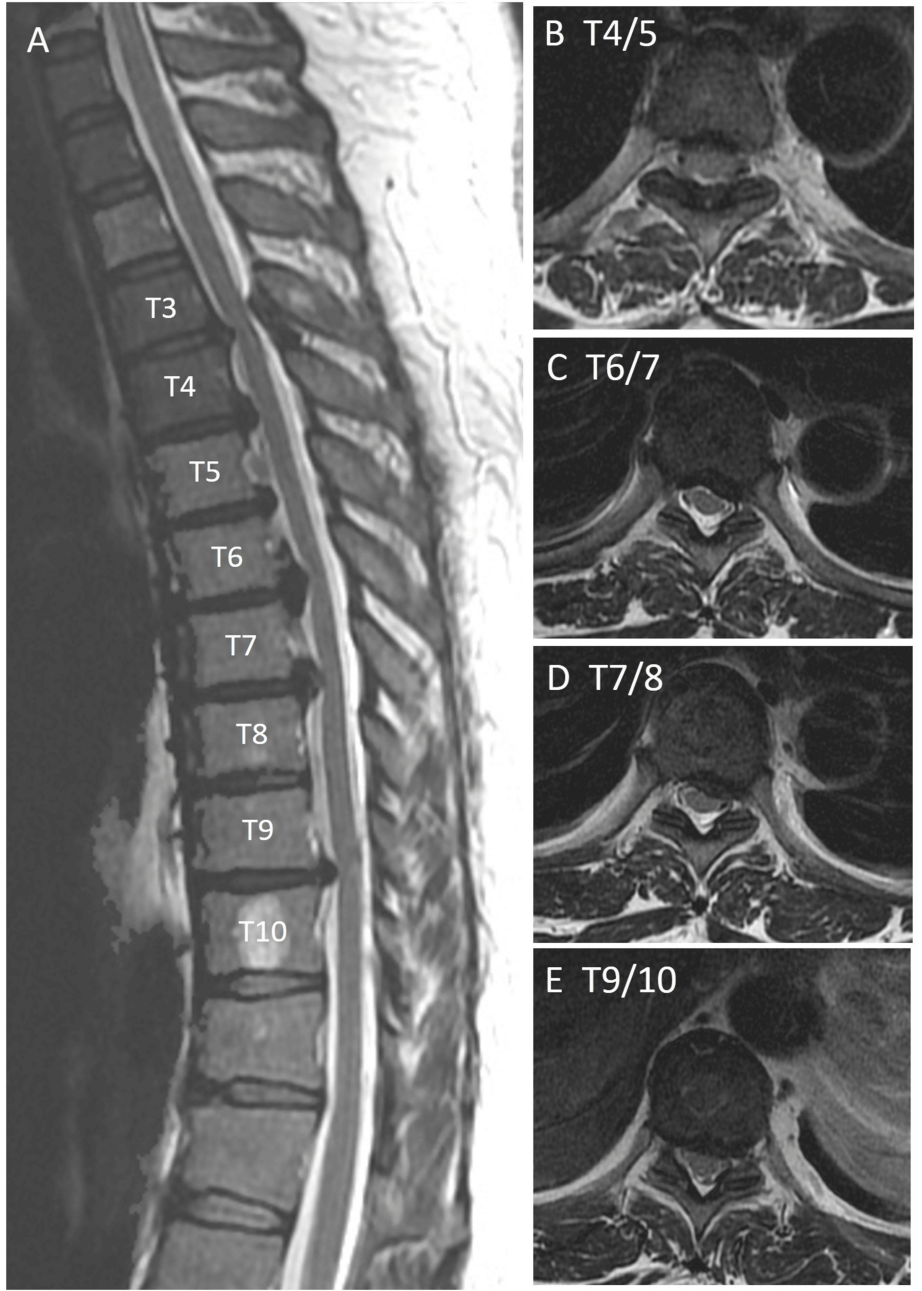
Preoperative T2-weighted MRI of the thoracic spine (A) Sagittal view. Segmental ossification of the posterior longitudinal ligament causing moderate spinal cord compression. (B) T4/5 level stenosis. (C) T6/7 level stenosis. (D) T7/8 level stenosis. (E) T9/10 level stenosis.

The patient received a botulinum toxin injection administered by a urologist, targeting the urethral region. Before surgery, spinal anesthesia was administered using bupivacaine (11 mg) at the subdural region of the L3-4 level without encountering any immediate complications. The administered anesthesia achieved a level extending up to T5. However, following the operation, the patient did not recover from spinal anesthesia and had complete paralysis of both lower limbs (Grade 0/5), and the anal sphincter tone was preserved. This paralysis did not improve on postoperative day 1. MRI of the thoracic and lumbar spine 12 hours after the procedure revealed OPLL spanning from T3 to T9, accompanied by severe moderate spinal stenosis (Figure 2).

**Figure 3:**
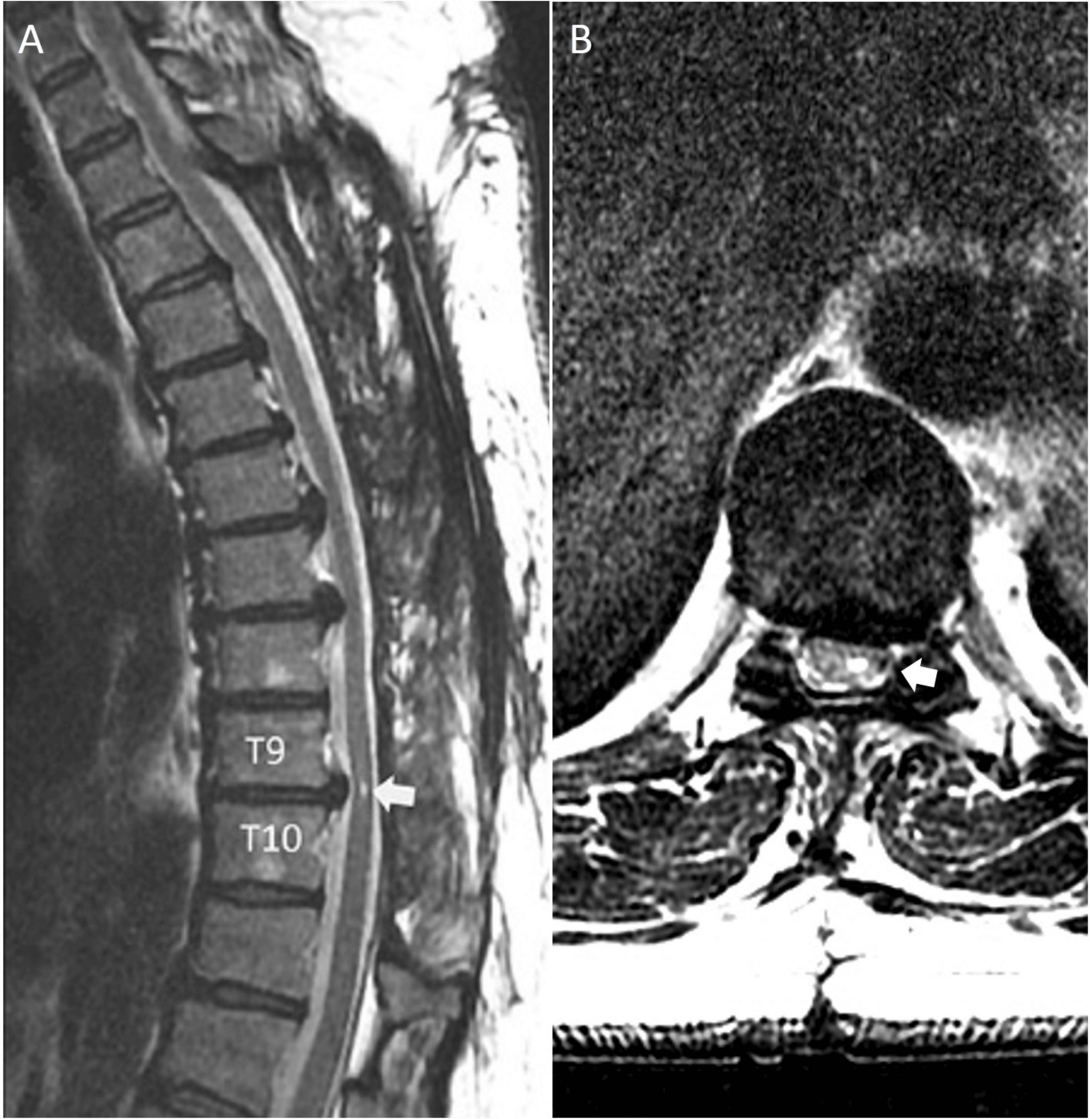
Postoperative two-year MRI of the thoracic spine revealed well decompression of the thoracic spine (A) Sagittal view. Myelomalacia (arrow) was noted at T9/10 level. (B) Axial view. T9/10 level. Myelomalacia (arrow) was noted.

Total laminectomy was performed between the T3 and T10 levels approximately 38 hours after the initial spinal anesthesia. The patient’s muscle power over the bilateral lower legs improved to Grade 1/5 immediately after the decompression surgery and Grade 3/5 1 day after the surgery. The patient was subsequently transferred to the rehabilitation department and could ambulate without assistance upon discharge. At two years postoperatively, the patient had muscle power Grade 5/5 of both lower limbs and was ambulatory with minimal numbness over the bilateral feet. The two-year follow-up MRI revealed well decompression of the thoracic spine, and focal myelomalacia was demonstrated at the level of T9-10 (Figure 3).

## Discussion

Lumbar puncture induces cerebral herniation in the presence of intracranial lesions with elevated intracranial pressure. Furthermore, in cases of spinal lesions accompanied by pre-existing stenosis, lumbar puncture can precipitate myelopathy. Several documented cases have shed light on this phenomenon. The initial case was reported by Eaton in 1940. Subsequently, Jooma introduced the term “spinal coning” in 1984 to characterize this phenomenon [2,3]. The reported cases encompassed patients with occult tumors, infections, and degenerative changes within the spinal canal [4]. Multiple mechanisms contribute to lumbar puncture-related paraplegia. Hollis proposed that an abrupt change in CSF pressure diminishes its protective buffering effect, thereby exacerbating the severity of stenosis [5]. Another suggested causative factor involves epidural venous engorgement, which is further compounded by a reduction in CSF pressure. This engorgement subsequently impairs venous drainage from the spinal cord below the level of compression, leading to cord swelling and increased compression [5].

The paraplegia observed in our patient is most likely attributed to spinal coning. First, the extended duration of symptoms cannot be accounted for by the brief analgesic effect [6]. Furthermore, the improvement in paraparesis following decompression surgery is of significant importance. Although no evident signs of myelopathy were observed before lumbar puncture (e.g., unsteady gait or numbness), the patient’s history of cervical spine OPLL suggests an increased predisposition to thoracic OPLL [7-9]. The literature indicates that 7-21% of patients with cervical OPLL present with thoracic OPLL. In a study by Park, among 68 patients who underwent cervical decompression for cervical OPLL, six had thoracic myelopathy necessitating further surgical intervention [7]. Therefore, the presence of a history of cervical OPLL is highly correlated with the likelihood of thoracic OPLL.

Because of the potential risk of spinal coning, spinal stenosis above the level of lumbar puncture has been proposed as a contraindication for the procedure [10]. The identification of patients with spinal stenosis before performing a lumbar puncture is essential. However, performing CT or MRI for every patient undergoing lumbar puncture is not practical. Therefore, a practical approach to mitigating the risk of spinal coning involves reviewing the patient’s medical history, performing a neurological examination, and considering relevant imaging studies in patients at risk of spinal stenosis.

## Conclusions

Surgeons and anesthesiologists should be aware of patients who may have pre-existing spinal stenosis, thus allowing them to avoid spinal coning and consequent complications. A patient's medical history, symptoms, and signs related to spinal stenosis should be carefully reviewed. Prompt identification of spinal coning and early surgical intervention lead to improved recovery.

## References

[1] Collier J (1904). The false localising signs of intracranial tumour. Brain.

[2] Eaton LM, Craig WM (1940). Tumor of the spinal cord: sudden paralysis following lumbar puncture. Proe Staff Meet Mayo Clin.

[3] Jooma R, Hayward RD (1984). Upward spinal coning: impaction of occult spinal tumours following relief of hydrocephalus. J Neurol Neurosurg Psychiatry.

[4] Morgan RJ, Steller PH (1994). Acute paraplegia following intrathecal phenol block in the presence of occult epidural malignancy. Anaesthesia.

[5] Hollis PH, Malis LI, Zappulla RA (1986). Neurological deterioration after lumbar puncture below complete spinal subarachnoid block. J Neurosurg.

[6] Ruppen W, Steiner LA, Drewe J, Hauenstein L, Brugger S, Seeberger MD (2009). Bupivacaine concentrations in the lumbar cerebrospinal fluid of patients during spinal anaesthesia. Br J Anaesth.

[7] Park JY, Chin DK, Kim KS, Cho YE (2008). Thoracic ligament ossification in patients with cervical ossification of the posterior longitudinal ligaments: tandem ossification in the cervical and thoracic spine. Spine (Phila Pa 1976).

[8] Liang H, Liu G, Lu S, Chen S, Jiang D, Shi H, Fei Q (2019). Epidemiology of ossification of the spinal ligaments and associated factors in the Chinese population: a cross-sectional study of 2000 consecutive individuals. BMC Musculoskelet Disord.

[9] Fujimori T, Watabe T, Iwamoto Y, Hamada S, Iwasaki M, Oda T (2016). Prevalence, concomitance, and distribution of ossification of the spinal ligaments: results of whole spine CT scans in 1500 Japanese patients. Spine (Phila Pa 1976).

[10] Costerus JM, Brouwer MC, van de Beek D (2018). Technological advances and changing indications for lumbar puncture in neurological disorders. Lancet Neurol.

